# Supplementary figures and images for: Cytochalasin B-Induced Membrane Vesicles from Human Mesenchymal Stem Cells Overexpressing IL2 Are Able to Stimulate CD8+ T-Killers to Kill Human Triple Negative Breast Cancer Cells
Source: Biology (Basel). 2021 Feb 10;10(2):141. doi: 10.3390/biology10020141 (PMC7916789; doi:10.3390/biology10020141)

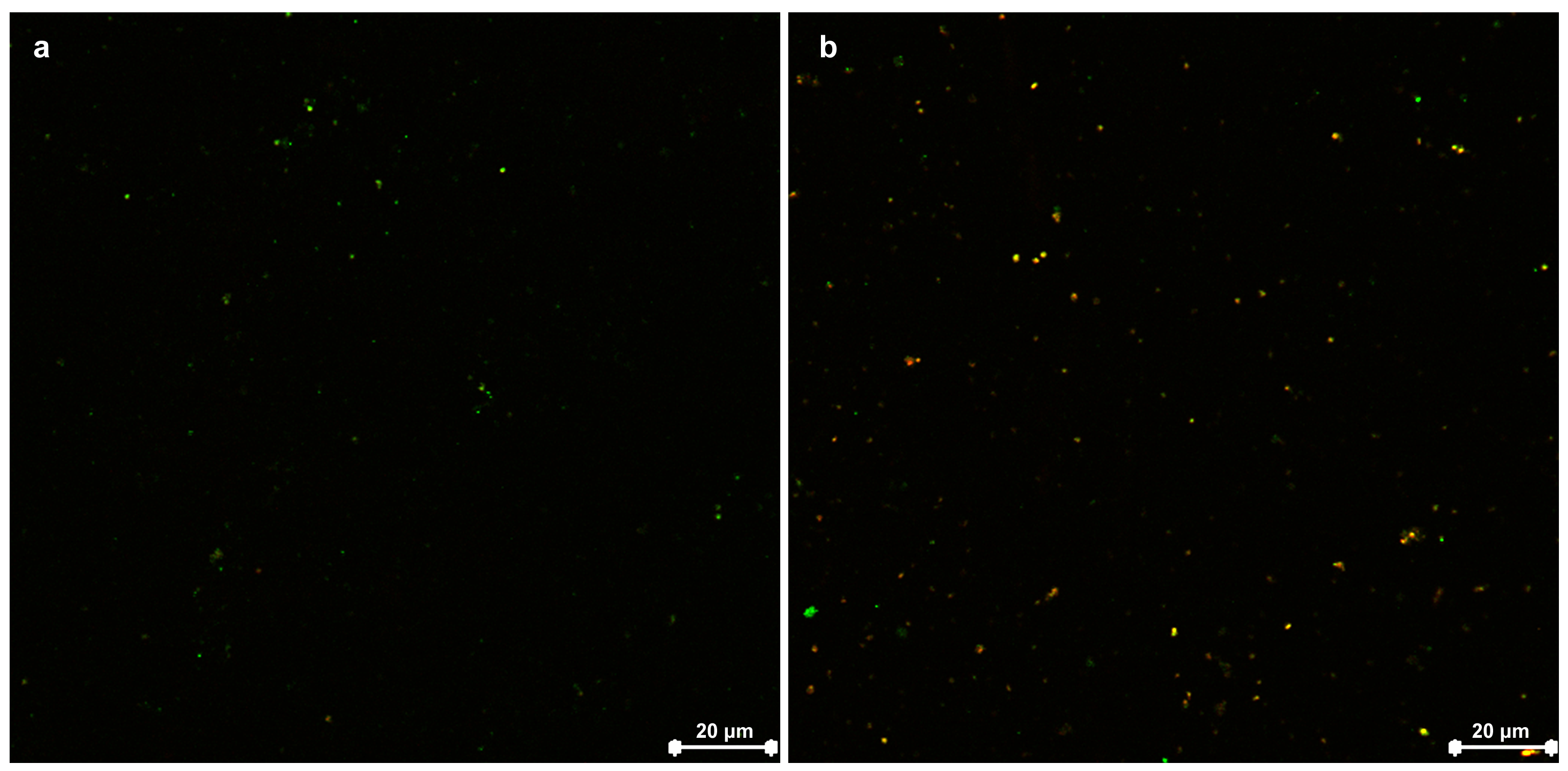

Supplement: Supplementary file 1 [file biology-10-00141-s001.zip › Supplementary Files/Figure S2.tif]

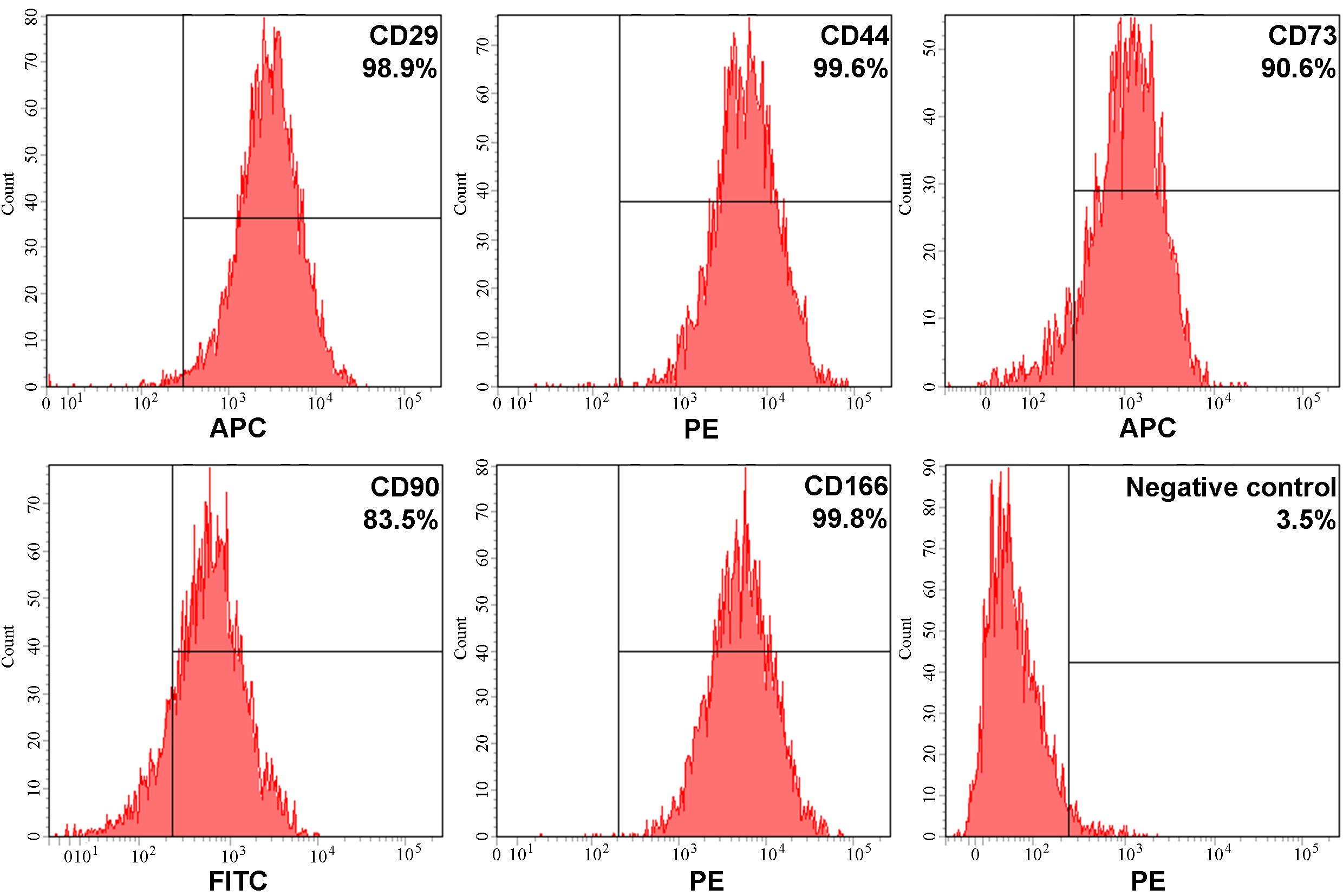

Supplement: Supplementary file 1 [file biology-10-00141-s001.zip › Supplementary Files/Figure S1.tif]
